# A research protocol for a pilot, randomized controlled trial designed to examine the feasibility of a dyadic versus individual yoga program for family caregivers of glioma patients undergoing radiotherapy

**DOI:** 10.1186/s40814-019-0479-5

**Published:** 2019-07-25

**Authors:** Kathrin Milbury, Jing Li, Shiao-Pei Weathers, Tina Shih, Smitha Malliaha, Yisheng Li, Lorenzo Cohen

**Affiliations:** 1 Department of Behavioral Science, The University of Texas MD Anderson Cancer Center, 1155 Pressler St, Houston, TX 77030 USA; 2 Department of Radiation Oncology, The University of Texas MD Anderson Cancer Center, Houston, TX USA; 3 Department of Neuro-Oncology, The University of Texas MD Anderson Cancer Center, Houston, TX USA; 4 Department of Health Services, The University of Texas MD Anderson Cancer Center, Houston, TX USA; 5 Department of Biostatics, The University of Texas MD Anderson Cancer Center, Houston, TX USA; 6 Department of Palliative, Rehabilitation & Integrative Medicine, The University of Texas MD Anderson Cancer Center, Houston, TX USA

**Keywords:** Family caregivers, Glioma, Dyads, Yoga, Feasibility, Randomized control trial, Study protocol, Quality of life

## Abstract

**Background:**

Although the diagnosis and treatment of a primary brain tumor present unique challenges to patients and their family caregivers, evidence-based supportive care interventions are generally lacking. The primary aim of this research protocol is to determine the feasibility of implementing a dyadic yoga (DY) versus a caregiver yoga (CY) intervention or a wait-list control (WLC) group using a randomized controlled trial design.

**Methods:**

Seventy-five glioma patients undergoing radiotherapy and their family caregivers are randomized to the DY, CY, or a WLC group. Patient-caregiver dyads in the DY group and caregivers in the CY group receive 15 sessions (45 min each) over the course of patients’ standard radiotherapy (6 weeks). Patients and caregivers in all groups complete baseline assessments of symptoms, quality of life (QOL), and health utilization outcomes prior to randomization. Follow-up assessments are performed 6 weeks and then again 3 months later. The primary outcome is feasibility (i.e., ≥ 50% of eligible dyads consent, ≥ 70% of enrolled dyads complete all assessments, and ≥ 50% of all practice sessions are attended). We will also perform primarily descriptive analyses of the self-reported outcomes (e.g., fatigue, overall QOL) and explore potential intervention moderators (e.g., performance status) to inform a larger future trial.

**Conclusion:**

This trial will provide important information regarding the feasibility of a dyadic versus a caregiver yoga intervention regarding symptom, QOL, and health utilization outcomes in glioma patients and their caregivers.

**Trial registration:**

ClinicalTrials.gov NCT02481349

## Background and rationale

While patients carry the brunt of the quality of life (QOL) sequelae associated with the diagnosis and treatment of cancer, the disease affects the whole family [1]. Family caregivers (FCGs) shoulder tremendous responsibility regarding offering emotional support and assistance with patients’ activities of daily living and help with managing their symptoms and other aspects associated with cancer treatment [2]. FCGs of patients with brain tumors such as glioma (80–85% of all brain tumors) are faced with unique challenges such as coping with personality changes and cognitive/neurological deficits the patient may experience due to the disease process and cancer treatment [3–15]. The emotional burden of coping with the uncertainty of the disease course and the general poor prognosis tends to be overwhelming [16]. Given the relatively short survival and poor QOL of glioma patients, it is not surprising that FCGs report high rates of distress, fatigue, and sleeping disturbances, which may undermine the quality of care they are able to provide to the patient [17–25]. In fact, FCGs may even experience significantly greater levels of anxiety and depression than glioma patients [7]. In addition to reduced QOL and increased symptoms, caregivers are at risk for increased morbidity (e.g., cardiovascular disease, diabetes, arthritis) and mortality, possibly related to their vulnerability to chronic stress, depression, and poor health behaviors/self-care [26–28]. Lastly, the financial burden for family members is high, both in outright expenses as well as lost income and benefits [2, 29, 30].

The field of behavioral cancer control is increasingly recognizing the need for supportive care interventions for caregivers. Based on a recent meta-analysis of randomized controlled trials (RCTs) in cancer, behavioral interventions yield small to medium effects in reducing caregiver burden and improving aspects of their QOL [31]. In addition to caregiver interventions, dyadic psychosocial interventions enrolling patients and caregivers jointly reveal small effects regarding improved cancer adjustment [32]. With a few exceptions, dyadic intervention studies have predominately enrolled patients with localized breast or prostate cancer [31–39]. In neuro-oncology, evidence-based supportive care interventions for both caregivers and patients (separate or together) are generally lacking. There is some evidence that, for patients, neurocognitive rehabilitation and neurorehabilitation is beneficial [40–42]. For FCGs of glioma patients, cognitive-behavioral therapy (CBT) may increase caregiving mastery and QOL [43]. These previous interventions show promise regarding feasibility and efficacy; however, the majority of them have a patient focus, so that caregivers are enrolled to assist patient coping or learn skills to better care for the patient [31, 39, 44]. Few interventions focus on the needs of the couple or family as a unit, and even fewer focus on the QOL and well-being of caregivers [31]. Moreover, it is unknown if caregivers are more likely to benefit from a caregiver-only intervention versus one that includes the patient. Given the shared cancer experience, a dyadic intervention may be advantageous because distress and QOL are interdependent (patient outcomes influence caregiver outcomes and vice versa) in families coping with cancer [45–49]. Thus, a dyadic intervention that offers supportive care in a holistic manner may optimize efficacy. According to evidence in the health-behavior literature, dyadic interventions may also be more feasible regarding participant retention and treatment adherence compared to individual programs [34, 50, 51].

Given the multifaceted needs of families coping with glioma [52–54], a behavioral intervention addressing both psychological and physical needs may be beneficial. Mind-body medicine such as yoga that integrates mindfulness training with various relaxation techniques and physical postures/exercises has been shown to improve cancer patients’ physical, psychological, and even cognitive symptoms [55–59]. Similarly to psychosocial interventions, the majority of yoga trials have included women with breast cancer. Two very small trials that enrolled family caregivers of cancer patients are published suggesting that further investigation of a yoga program for caregivers is warranted [60, 61].

### Objectives

To address the gaps in the literature regarding supportive care interventions for families coping with a primary brain tumor, we developed a yoga program to reduce symptoms and increase QOL outcomes. This research builds upon our previous pilot studies examining the feasibility and preliminary evidence for efficacy of a dyadic yoga intervention for patients with glioma undergoing radiotherapy (RT) and their FCG [62, 63]. We now seek to collect data on the feasibility of implementing a pilot RCT of a patient-caregiver dyadic yoga versus a caregiver yoga intervention including the administration of self-reported QOL and health utilization measures. Additionally, we will obtain descriptive evidence of self-reported outcomes to inform a subsequent clinical trial.

In summary, our specific aims are to

1. Determine the feasibility of implementing an RCT for FCG and patients with glioma involving a dyadic yoga (DY), a caregiver yoga (CY), and a usual care wait-list control (WLC) group.
2. Perform descriptive analyses of QOL outcome measures in patients and their FCGs.

As an exploratory aim, we will carry out descriptive analyses including correlations between symptom, QOL, and health utilization measures and measures of potential moderation (e.g., baseline psychological distress, patients’ tumor grade, and performance status) to potentially identify subgroups who reveal a differential response to the yoga interventions.

## Methods

### Design overview and study setting

The design will involve a feasibility randomized controlled trial with parallel allocation. Caregiver-patient dyads will complete the initial self-report assessments, and then be randomized to the DY, CY, or WLC groups. Follow-up assessments will be completed within 1 week of finishing RT and again 3 months later. For each completed questionnaire, participants will receive a $20 gift card ($60 per participant). Participants’ parking costs for yoga session appointments will be paid if they are not covered by the hospital. Participants in the DY and CY intervention groups will attend 15 sessions (60 min each) over the course of patients’ 6-week standard RT (2–3 sessions per week). Dyads in the WLC group will receive usual care. Sessions 1–4 are delivered in person in a room designated for behavioral interventions, which is located near the RT treatment area. For sessions 5–15, participants will have the option to continue to attend the sessions at the hospital or receive the sessions via HIPAA compliant videoconferencing delivery (e.g., Facetime, Zoom). For participants who have internet access but no electronic device and would like to use videoconferencing, we will loan an iPad for the course of the intervention period.

### Eligibility

#### Inclusion criteria

Both family caregiver (e.g., spouse/partner, parent, adult child, sibling) and patient must be willing to participate in this study and must be (1) at least 18 years old, (2) able to read and speak English, and (3) able to provide informed consent. Patients must be (1) diagnosed with primary malignant glioma, (2) scheduled to undergo daily RT for 6 weeks, and (3) have a Karnofsky Performance Status (KPS) of 80 or more.

#### Exclusion criteria

Participants will be excluded if they have regularly (self-defined) practiced yoga in the year prior to patient’s diagnosis. Patients with cognitive deficits that would impede the completion of self-report instruments as measured by the Mini-Mental State Examination (score of 17 and below) will be excluded.

### Interventions

#### Dose

Participants in both groups will receive practice sessions 2-3 days per week over the course of standard 6-weeks RT (15 sessions total, 45 min each).

#### DY group

Patient and FCGs will attend all sessions together. The content was developed in collaboration with Vivekananda Yoga Anusandhana Samsthana (VYASA) Yoga Research Foundation, in Bangalore, India, and specifically designed around the needs and limitations for brain tumor patients. We acceptability tested the intervention content in our previous work [63, 64]. The DY program includes a brief introduction to the dyadic program followed by individual and dyadic mind-body techniques. Following a universal Hatha Yoga practice, the program consists of four main components: (1) joint loosening with mindfulness training (e.g., breath synchronization), (2) postures (asanas) with deep relaxation techniques, (3) breathing exercises (pranayama) with sound resonance, and (4) meditation. Because of contraindications (e.g., avoid “head-below-heart”), common yoga postures (i.e., forward bend and downward-facing dog) are excluded. Instead, we selected several postures (e.g., tree pose, triangle) to help with balance disturbances that are common in glioma patients as well as older adults. Sessions 1–4 will focus on gradually introducing the various practices. Once participants have learned the techniques, the rest of the sessions (5–15) will focus on refining the practice, answering questions related to the practice and discussing participants’ experiences. Each session will start with an introduction/review of the previous session, an intention for the practice (e.g., identify symptoms/concerns, goals for the practice) followed by the mind-body techniques building upon the content previously learned. Practices will include available modifications based on participants’ physical limitations, and participants’ safety is ensured at all times.

The underlying philosophy of the DY program is based on the principals of interdependence: mutual and reciprocal support, teamwork, and togetherness. These principals are interwoven in all aspects of the program. We will incorporate dyadic postures, physical touch (e.g., holding hands, partner stretches, and breathing exercises), and meditations that focus on dyadic concepts (e.g., emotional closeness, acceptance of self and partner, and compassion for self and partner). Starting with session 1, the instructor will communicate that the practice is intended to target the needs of both members of the dyad with a focus on their interconnectedness and togetherness while going through the cancer experience.

#### CY group

This group will follow a traditional yoga practice in which delivery and content focuses on the individual, consistent with the vast majority of yoga programs. Intervention procedures will be similar to the DY group; however, caregivers will be instructed without the patient, and all content will focus on the caregiver, including individual coping and QOL management skills to practice self-care. Session structure and content is will be as similar to the dyadic format as possible with the exception of the dyadic focus, partner exercises, and meditations. To avoid contamination of patients, partners in the CY group will be asked not to share study content with others including the patient for the duration of the study.

#### Home practice

Participants in both yoga groups will receive a DVD, URL information to access electronic video files, and printed materials and will be encouraged to practice together (DY group) or individually (CY group) on days when they do not meet with the instructor and asked to keep a practice diary to be turned in on a weekly basis. Participants in the CY group are asked on a weekly basis if patients participated in their home practice to assess for patient contamination.

#### WLC group

Participants in the WLC group will receive usual care as provided by the MD Anderson health care team for cancer patients and will complete all assessments during the same time frame as the DY and CY intervention groups except for the intervention evaluations. After the last assessment, they will be offered the intervention based on their preference but no additional assessments will be collected.

#### Interventionist training and quality control

Both yoga groups will be taught by at least two certified (International Association of Yoga Therapists; C-IAYT) yoga instructors with at least 5 years of experience in working with cancer patients and caregivers to reduce therapist-specific effects. Instructors will receive an instructor’s version of the yoga manuals including scripts and scenarios and role-play with members of the research team before implementing the DY and CY interventions. To ensure treatment fidelity, all sessions for both yoga groups will be audio and video recorded with the participants’ permission (obtained during the informed consent process). Using a fidelity checklist, 10% of randomly selected recordings will be reviewed by a researcher (KM) to ascertain whether each intervention component was appropriately delivered and whether any extraneous material was included. The sessions will be reviewed on an ongoing basis so that feedback can be provided to the interventionists as necessary.

### Study measures

#### Demographic and medical factors

Both patients and caregivers will be asked for demographic information including age, sex, race/ethnicity, marital status/length, occupational status, and educational history. Patient medical information regarding disease and treatment factors will be obtained from their medical records.

#### Feasibility

Consent rates including refusal reasons, study attrition, class attendance, and completion of each questionnaire will be documented. We will document if participants attended sessions 5–15 at the hospital or via videoconference delivery including reasons for their preference. For those choosing videoconference delivery, we will record if they use their own or a loaner device. Participants in the yoga arms will be asked to complete paper-pencil practice logs of their home practice, and if they practiced together or alone (dyads in the DY group) and shared the intervention content with others (CY group), which will be submitted to the instructor on a weekly basis. After the last session is completed, using Redcap, participants in the yoga arms will be asked to identify aspects of the program they like or dislike, identify what they find most and least useful, communicate whether they perceive any benefit from the yoga program, and rate their interventionist.

#### Self-reported measures

Study staff involved in data collection will be blinded to the treatment conditions. Self-report measures were chosen based on their demonstrated psychometric properties, intervention targets, relevance to the targeted population, brevity, and clinical benchmarks. Both patients and caregivers in all groups complete self-reported measures assessing:

1. *Overall QOL* with the Medical Outcomes Study 36-item short-form survey (SF-36) assessing eight distinct domains: physical functioning, physical impediments to role functioning, pain, general health perceptions, vitality, social functioning, emotional impediments to role functioning, and mental health [65]. The measure yields a mental and physical composite summary (MCS and PCS, respectively).
2. *Depressive symptoms* will be assessed with the Centers for Epidemiological Studies–Depression measure (CES-D) [66]. The CES-D consists of 20 items focusing on the affective component of depression.
3. *Fatigue* with the Brief Fatigue Inventory (BFI) [67], a nine-item questionnaire designed to be used in the clinical setting to rapidly assess fatigue severity. The items range from 0 to 10, with 0 being “no fatigue” and 10 being “fatigue as bad as you can imagine,” and participants will rate their fatigue at its “worst” and “usual” and as it is “now.”
4. *Sleep disturbances* using the Pittsburgh Sleep Quality Index [68], an 18-item self-rated questionnaire that assesses quality of sleep and sleep disturbances.
5. *Health care utilization* will be assessed with a modified version of the National Health Interview Survey (NHIS) Questionnaire–Adult Access to Health Care & Utilization Questionnaire [69]. For patients, we will also obtain via medical records their utilization of various types of health care resources (e.g., hospitalizations, office visits) during the study period. *Productivity loss* will be measured by the Work Productivity and Activity Impairment General Health (WPAI-GH) [70] assessing presence at the work place [71]. Construct validity and test-retest reliability of the WPAI-GH have been established [70, 72]. Participants will complete items pertaining to productivity loss due to their own and FCG also complete it due to the patient’s health. Following Grosse et al [73], our estimation of economic productivity will include two components: market and household productivity. Market productivity captures lost wages from labor market activities (absenteeism and presenteeism) whereas household productivity is quantified as the value of time spent at household activities, such as child care, which are especially relevant to individuals who are not in the labor market.

### Sample size and sample size considerations

We will randomize 75 dyads to either the DY intervention, CY, or WLC groups (25 dyads per group). Based on Whitehead et al.’s (2016) recommendation, a pilot trial of 25 participants per group will detect a small standardized effect size with 90% power and two-sided 5% significance [74]. Of note, this trial is not designed to examine intervention efficacy, and we have not carried out any formal power calculations. In line with recommendations in the literature, we focus on examining feasibility and descriptive analyses [75–77]. Moreover, a sample size of 75 dyads is consistent with previously published psycho-oncological feasibility trials, in which the investigators typically enrolled 20–25 participants/dyads per study group [78].

### Recruitment

Research staff will identify potential participants via the Institution’s computerized appointment system. We will approach potential participants during their initial RT consult or RT simulation visits (prior to starting RT), screen them for eligibility, and ask them for consent. If a patient’s caregiver is not present during the initial contact, we will ask the patient for permission to contact the caregiver via telephone to obtain consent.

### Randomization

Dyads will be randomized using minimization [79], which is similar to stratified randomization in that participant characteristics are used to assign participants to a study condition. However, minimization results in better group balance and does not suffer from some of stratification’s limitations (e.g., increased probability of group imbalance when stratifying across several characteristics). Factors will include patient and caregiver sex and age and patient tumor grade (LGG; HGG) and KPS (100; 90; 80).

### Blinding

This will be an unmasked trial. However, research staff involved in data collection will be blind to group allocation.

### Data collection

Dyads in all three groups will be assessed at three time points: baseline, 6 weeks later, and 3 months later. All assessments are collected via online surveys (Redcap).

### Statistical methods

To evaluate our study aims and determine whether the DY and CY interventions should be further evaluated in a subsequent larger trial, we will follow the steps described below.

#### Specific aim 1

The primary objective of this research is to determine feasibility according to overall accrual, attrition, completion of questionnaires, and session adherence. We will calculate rates, frequencies, and 90% confidence intervals when applicable and judge the trial to be feasible if at least 50% of eligible dyads consent to participate (i.e., approach 150 couples to accrue 75). Moreover, we will deem the trial feasible if at least 70% of enrolled dyads (≥ 54) are retained and complete all follow-up assessments and on average at least 50% of all practice sessions are attended.

#### Specific aim 2

We will perform descriptive analyses of symptoms and QOL measures for patients and their FCGs, including calculations of means, standard deviations, and confidence intervals.

#### Exploratory aim

As an exploratory aim, we will explore descriptive analyses including cross-sectional and prospective correlations of measures of medical and demographic factors (e.g., grade and age) and psychosocial factors (e.g., baseline CES-D scores) [80]. We will also explore a dose effect examining if class attendance and home practice are associated with QOL measures. We will explore if delivery (i.e., hospital versus videoconferencing) is associated with differential outcomes. These results will inform optimal dosing of the yoga program in the larger study. Additionally, we will calculate the intraclass coefficients for patient and FCG variables to further provide a rationale for this dyadic intervention.

## Discussion

This study will provide the necessary evidence of the feasibility of an RCT comparing a dyadic yoga with a caregiver yoga intervention relative to a usual care WLC group in patient-caregiver dyads coping with glioma, a vulnerable yet understudied population. Given the paucity of existing RCTs of behavioral supportive care interventions for patients with advanced cancer, findings of this research will be of interest to other researchers working in this area or wanting to examine recruitment for other intervention studies in similar populations. Determining participants’ preference regarding intervention delivery (at the hospital versus videoconferencing) will inform the design and feasibility of other behavioral intervention research.

We acknowledge that the proposed study sample excludes patients not receiving standard RT and other types of brain tumors. Given that this is a small trial, we believe that a rather homogenous sample is justifiable at this stage of development. However, if our feasibility criteria are not met, we will expand the trial to patients and their family caregivers in later time points in the treatment trajectory. We do not propose to examine effect sizes because they may be misleading [75–77]. If the trial will be deemed feasible, the clinically meaningful changes in study outcomes examined here (if any) will inform the sample size of a future larger trial. We will present findings of this feasibility trial at national and international behavioral medicine and supportive care conferences and submit them for publication in peer-reviewed journals.

### Trial status

Participant recruitment started on May 2018 and is expected to be finished by April 2020.

## Data Availability

The final anonymized trial dataset resulting from this study will be available to other researchers upon request from the corresponding author.

## Abbreviations

C-IAYT: International Association of Yoga Therapists
CY: Caregiver yoga
DY: Dyadic yoga
HIPAA: Health Insurance Portability and Accountability Act
QOL: Quality of life
RCT: Randomized controlled trial
WLC: Wait-list control
